# Iron Salts, High Levels of Hemoglobin and Ferritin in Pregnancy, and Development of Gestational Diabetes: A Systematic Review

**DOI:** 10.1055/s-0042-1755460

**Published:** 2022-09-06

**Authors:** Vanessa Iribarrem Avena Miranda, Tatiane da Silva Dal Pizzol, Patricia Romualdo de Jesus, Marysabel Pinto Telis Silveira, Andréa Dâmaso Bertoldi

**Affiliations:** 1 Post-Graduate Program in Epidemiology, Federal University of Pelotas, Pelotas, Brazil; 2 Post-Graduate Program in Epidemiology, Federal University of Rio Grande do Sul, Porto Alegre, RS, Brazil; 3 Institute of Biology, Department of Physiology and Pharmacology and Post-Graduate Program in Epidemiology, Federal University of Pelotas, Pelotas, Brazil

**Keywords:** ferritin, gestational diabetes, hemoglobin, iron supplement, pregnancy, ferritina, diabetes gestacional, hemoglobina, suplemento de ferro, gravidez

## Abstract

**Objective** The aim of this study was to systematically review literature on the use of iron supplements (not including iron derived from diet), increased levels of hemoglobin and/or ferritin, and the risk of developing gestational diabetes mellitus (GDM).

**Data source** The following databases were searched, from the study's inception to April 2021: PUBMED, Cochrane, Web of Science, Scopus, Embase, Cinahl and Lilacs.

**Selection of studies** A total of 6,956 titles and abstracts were reviewed, 9 of which met the final inclusion criteria, with 7,560 women in total.

**Data collection** Data extraction was performed by two independent reviewers and disagreements were resolved by a third researcher.

**Data synthesis** Methodological quality in controlled trials were assessed according to the Cochrane Collaboration tools (ROB-2 and ROBINS-1) and for the observational studies, the National Institutes of Health's (NIH) quality assessment tool was used. Among the 5 observational studies, women with a higher hemoglobin or ferritin level were more likely to develop GDM when compared with those with lower levels of these parameters. Among the 3 randomized clinical trials, none found a significant difference in the incidence of GDM among women in the intervention and control groups. However, we identified many risks of bias and great methodological differences among them.

**Conclusion** Based on the studies included in this review, and due to the important methodological problems pointed out, more studies of good methodological quality are needed to better establish the association between iron supplementation and GDM.

## Resumo

**Objetivo** O objetivo deste estudo foi revisar sistematicamente a literatura sobre o uso de suplementos de ferro (não incluindo o ferro derivado da dieta), aumento dos níveis de hemoglobina e/ou ferritina e o risco de desenvolver diabetes mellitus gestacional (DMG).

**Fontes dos dados** as bases de dados PUBMED, Cochrane, Web of Science, Scopus, Embase, Cinahl e Lilacs foram pesquisadas até abril de 2021.

**Seleção dos estudos** Foram revisados 6.956 títulos e resumos, dos quais 9 preencheram os critérios finais de inclusão, totalizando 7.560 mulheres.

**Coleta de dados** A extração de dados foi realizada por dois revisores independentes e as divergências foram resolvidas por um terceiro revisor.

**Síntese dos dados** A qualidade metodológica dos ensaios controlados foi avaliada de acordo com as ferramentas da Colaboração Cochrane (ROB-2 e ROBINS-1) e para os estudos observacionais, foi utilizada a ferramenta de avaliação de qualidade do National Institutes of Health (NIH). Entre os 5 estudos observacionais, as mulheres com maiores níveis de hemoglobina ou ferritina apresentaram maior probabilidade de desenvolver DMG quando comparadas àquelas com níveis mais baixos nesses parâmetros. Entre os 3 ensaios clínicos randomizados, nenhum deles encontrou diferença significativa na incidência de DMG entre as mulheres dos grupos de intervenção e controle. No entanto, identificamos muitos riscos de viés e enormes diferenças metodológicas entre eles.

**Conclusão** Com base nos estudos incluídos nesta revisão e devido aos importantes problemas metodológicos apontados, são necessários mais estudos de boa qualidade metodológica para melhor estabelecer a associação entre suplementação de ferro e DMG.

## Introduction

Gestational diabetes mellitus (GDM) is a temporary condition characterized by hyperglycemia, which occurs due to glucose intolerance, with onset during pregnancy and usually disappearing shortly after delivery. 1 This condition is responsible for several maternal-fetal health consequences, such as the increased risk of malformations, fetal loss, and neonatal, perinatal, and maternal mortality. 2 In the long term, the maternal effect of GDM is the increased risk of metabolic syndrome and type 2 diabetes. In children, it almost doubles the risk of developing childhood obesity and metabolic syndrome compared with children born from non-diabetic mothers. 3

The prevalence of GDM is increasing globally, in parallel with the increase in type 2 diabetes mellitus and female obesity. 1 However, there is no accurate estimate of the overall incidence of gestational diabetes because screening and diagnostic patterns are not uniform throughout the world and over the years. 3 Estimates from the American Diabetes Association (ADA) indicate a prevalence of 1 to 14%. 4 However, in 2013, the World Health Organization (WHO) generated new estimates from the update of its new criterion, where the prevalence ranged from 4 to 25%, with more than 90% occurring in low- and middle-income countries. 1 5

The pathogenesis of GDM is unclear, although some authors suggest it is a complex disease with a combination of genetic and environmental factors. 6 According to the Diabetes Canada Clinical Practice Guidelines Expert Committee, risk factors increasing the risk of GDM include increased maternal age, a high-risk group (African, Arab, Asian, Hispanic, Indigenous, or South Asian), corticosteroid medication, obese (body mass index, BMI > 30 kg/m2), prediabetic, prior history of GDM, family history of diabetes (parent, brother or sister), polycystic ovary syndrome or acanthosis nigricans. 7 The American Diabetes Association includes other risk factors, such as overweight in current pregnancy, central deposition of body fat, short stature (< 151 cm), excessive fetal growth, polyhydramnios, hypertension, or preeclampsia in the current pregnancy, as well as a history of fetal or neonatal death, and macrosomia. 4

From the 2000s onwards, several authors have hypothesized that iron supplementation in women with normal levels of hemoglobin and serum ferritin, a practice recommended by several entities to prevent gestational anemia, may contribute to an increased risk of GDM. 8 9 10 The glucose metabolism can be affected by iron accumulation in several ways. One of the suggested mechanisms is regarding the high oxidation capacity of iron. Iron promotes the formation of hydroxyl radicals that can attack β-cell membranes and affect insulin synthesis and secretion in the pancreas. 11 However, some studies have not confirmed this hypothesis. 12 13 14 15 16 The role of iron excess in the pathogenesis of GDM needs to be examined, specifically to regard the iron provided by supplements. The present systematic review was conducted to answer the following question: “Does the use of iron supplements (not including dietary iron) and high levels of hemoglobin and/or ferritin increase the risk of developing GDM in non-anemic women?”

## Methods

A systematic review was performed according to the Preferred Reporting Items for Systematic Review and Meta-Analyzes (PRISMA). 17 The review protocol was registered in the international database PROSPERO, under the number CRD42018086269.

Experimental or observational studies in humans were included, with GDM as primary or secondary outcome, and the exposure being use of iron supplement during pregnancy (not including iron derived from diet), and the presence of at least one biochemical parameter of iron (hemoglobin or serum ferritin). Animal studies, uncontrolled studies, reviews, meta-analysis, protocols, abstracts, charts, and those that measured exposure through dietary iron intake or supplementary iron intake in all groups of pregnant women regardless of hemoglobin level were excluded.

The systematic review of the literature was performed in PUBMED, Cochrane, Web of Science, Scopus, Embase, Cinahl, and Lilacs databases, from inception to April 2021. No limits for year of publication and language were used. The gray literature was accessed through Capes Thesis database, the gray literature report ( http://www.greylit.org ) and Open Gray ( http://www.opengrey.eu ). The references of the included studies were reviewed to check for other possible studies to be included. For further details regarding search strategies for all databases, please refer to the supplementary file.

Three authors independently screened the titles and abstracts of all retrieved studies. Then full-text screening of each potentially eligible study was done independently by the authors, and discrepancies were resolved by a third reviewer. The online tools Covidence (Covidence Melbourne, Australia) and Rayyan(Qatar Foundation, Qatar) were used for article selection. 18

The following data were extracted from the eligible articles: country of origin, year of publication, design, health service used, sample size, iron dose (duration and frequency), age, number of women with GDM, gestational week of GDM diagnosis, biochemical parameters of iron, and gestational trimester of collection of these parameters. Methods to control for confounders and covariables such as parity, age, and BMI were recorded. The authors were contacted when additional information was needed. Of the 16 authors we contacted by e-mail, 4 responded. Two of our authors independently extracted data using a standardized data extraction form, and discrepancies were resolved by a third reviewer. The outcome of interest, GDM, was measured differently in the studies, and the authors' data was extracted as reported.

The risk of bias was independently assessed by two reviewers and the disagreements were resolved by a third reviewer. Risk of bias in randomized controlled trials (RCTs) was assessed according to the revised Cochrane risk of bias tool for randomized trials (RoB-2), 19 which recommended the evaluation of the following domains: risk of bias arising from the randomization process, bias due to deviations from the intended interventions, bias due to missing outcome data, bias in the measurement of the outcome, and bias in the selection of the reported result. The risk of bias for each domain assessed was classified as low, some concerns, and high. To evaluate the risk of bias in non-randomized controlled trials (NRSIs), we used the Risk Of Bias In Non-Randomized Studies of Interventions (ROBINS-I) tool, 20 with seven domains: bias due to confounding, bias in the selection of participants into the study, bias in classification of interventions, bias due to deviations from intended interventions, bias due to missing data, bias in the measurement of outcomes, and bias in the selection of the reported result. The risk of bias of domains was classified as low, moderate, serious, critical, and no information.

For the cohort and case-control studies, the NHS's 21 Quality Assessment Tool was used, with 14 questions for cohort and cross-sectional studies, and another one with 12 questions for case-control studies, with each item being rated as either yes (criterion met), no (criterion not met), not applicable, cannot determine, or not reported. The results of the analysis of the risk of bias were presented in figures using the Risk of bias VISualization (robvis) tool. 22

The main measures to summarize our outcome of interest were obtained as reported by the authors (e.g., relative risk, odds ratio, mean difference). Due to the variation in the study design, participants, interventions, and measures of results reported, it was decided to describe the studies, their results, applicability, and limitations in a qualitative synthesis, instead of performing a meta-analysis.

## Results

**Fig. 1:**
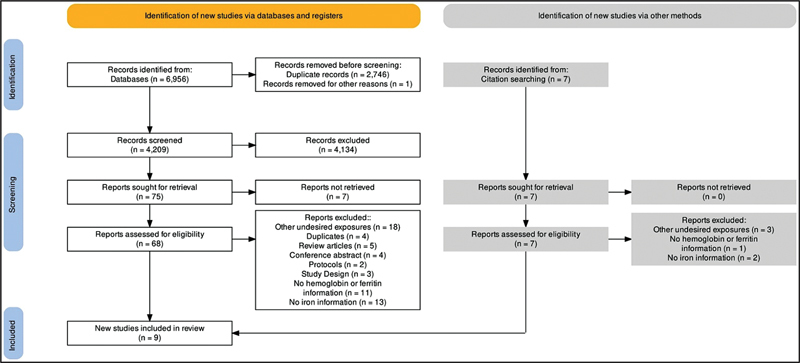
The PRISMA flowchart of search strategy and selection process.

Of a total of 6,956 studies identified, 4,209 were screened after exclusion of duplicates. After reading the titles and abstracts, 75 manuscripts were selected for a full reading, and of these, 7 reports could not be retrieved. Most studies were ruled out for other undesired exposures, such as supplementation in all groups of pregnant women, or no data on iron supplementation. After reading the articles and reviewing references, 9 studies were included in the review ( Fig. 1 ).

The characteristics of the included articles were described in Charts 1 and 2 . The selected works included cohort studies ( *n* = 3), 23 24 25 case controls ( *n* = 2), 26 27 and controlled trials ( *n* = 4). 13 28 29 30 The studies were conducted in the United Arab Emirates ( *n* = 1), China ( *n* = 4), Finland ( *n* = 1), Iran ( *n* = 1), Turkey ( *n* = 1), and the United States ( *n* = 1). Overall, 7,560 pregnant women participated in the studies, ranging from 58 to 3,289. Participants came from hospitals, 13 23 24 28 30 clinical centers, 27 polyclinics, 26 and primary health care settings. 29 One study did not report this data ( Chart 1 and 2 ). 13

**Chart 1:** Characteristics of the controlled trials included in the systematic review

First author (year) Country	Study design	Health service	GDM diagnosis: method/gestational week	Control group			Experimental group		
				N	Age	Iron dose (frequency, duration, trimester)	n	Age	Iron dose (frequency, duration, trimester)
Asadi (2019) 28 * Iran	NRCT	Hospital	NR	30 30	29.6 ± 12.3 27.5 ± 8.3	NR	30	29.6 ± 16.1	NR
Chan (2009) 13 China	RCT	Hospital	OGTT/ 28–30 and 36 weeks	599	31.3 ± 0.18	Placebo at minimum of 12 weeks (from 16–28), 1 ^st^ trimester	565	31.3 ± 0.19	60 mg EI daily, minimum of 12 weeks (16–28 weeks), 1 ^st^ trimester
Kinnunen (2016) 29 Finland	RCT	Primary health care	NR (diagnosis obtained through medical records) a	1,358	27.8 ± 5.6	50 mg EI twice a day, only if Hb fell below 100 g/L, 2 months or until their Hb increased to 110 g/L, 2 ^nd^ trimester	1,336	27.7 ± 4.9	100 mg EI daily b until delivery, all trimesters
Ouladsahebmadarek (2011) 30 Arab Emirates	RCT	Hospital	NR	372	25.48 ± 4.96	Placebo, 13 weeks, 1 ^st^ trimester	410	26.28 ± 5.25	30 mg EI daily, 13 weeks, 1 ^st^ trimester

**Abbreviations:** CI, confidence interval; EI, elemental iron; GDM, gestational diabetes mellitus; Hb, Hemoglobin; NR, not related; NRCT, non-randomized clinical trial; OR, odds ratio; RCT, randomized controlled trial.

**Notes:** *This study had three groups of comparison: women with normal serum ferritin levels (= 30 µg/dL) who received standard prophylactic iron supplementation during the pregnancy (designed as the experimental group); those who had minor thalassemia and El-evated serum ferritin levels (= 30 µg/dL) who did not receive prophylactic iron supplementation, or those with normal ferritin levels (≥ 30 μg/dL) who refused to take iron supplementation due to gastrointestinal upset (designed as control group 1); and those with iron deficiency anemia with low serum ferritin levels (< 30 μg/dL) who received standard iron supplementation during pregnancy (designed as control group 2).

a The authors stated that GDM “was not assessed systematically among all participants, but was abstracted from patient records as recognized in the usual care.”

b The participants received 100 mg elemental iron throughout the pregnancy regardless of Hb level.

**Chart 2:** Characteristics of the observational studies included in the systematic review. Cohort study ( *n* = 3) and case-control ( *n* = 2)

First author (year) Country	Study design	Health service	GDM diagnosis: method/ gestational week	Sample size					Age	Iron dose (frequency, duration)
				N	Control group	Exposed group	Case group	Control group		
Özyiğit (2008) 26 Turkey	Case-control	Polyclinic	OGTT/ 24–28 weeks	58	23	35	−	−	26	40 mg IS daily for at least 2 months
Rawal (2016) 27 United States	Case-control	Clinical centers	NR (diagnosis obtained through medical records)/ 10–14 and 15–26 weeks	321	−	−	107	214	Control group: 30.4 ± 5.4 Case group: 30.5 ± 5.7	Dose: NR 83% at weeks 10–14; 87% in the 2 ^nd^ trimester during 15–26 gestational weeks reported using IS
Liu and Pang (2018) 23 China	Cohort	Hospital	NR/at delivery	259	124	135	−	−	Control group 31.1 ± 1.5 Exposed group 30.9 ± 1.3	300 mg of IS daily, 7 days weekly until the baby delivery
Zhu (2019) 25 China	Cohort	NR	OGTT 24–28	3,289 ^a^	1 ^st^ 2,508 2 ^nd^ 2,805	1 ^st^ 777 2 ^nd^ 433	−	−	26.4 ± 3.7	NR
Si (2020) 24 China	Cohort	Hospital	OGTT/ 24–28	1,128 ^b^	1 ^st^ 831 2 ^nd^ 639	1 ^st^ 297 2 ^nd^ 489	−	−	GDM group 28.60 ± 3.33 No GDM group 27.91 ± 3.25	NR

**Abbreviation:** GDM, gestational diabetes mellitus; IS, iron supplementation; NR, Not related; OGTT, oral glucose tolerance test. **Notes:** ^a^ The 3,289 pregnant women were evaluated in the 1 ^st^ and 2 ^nd^ trimesters of pregnancy, with 4 missing in the 1 ^st^ and 51 missing in the 2 ^nd^ trimester; ^b^ The 1,128 pregnant women were evaluated in the 1 ^st^ and 2 ^nd^ trimesters of pregnancy.

In the controlled trials, the daily dose of iron supplementation ranged from 30 to 100 mg of elemental iron in the experimental groups. 13 29 30 In the observational studies, one study reported 40 mg/day of elemental iron 26 and another 300 mg daily. 23 The other studies did not report this data. 24 25 27 28 The duration of supplementation throughout pregnancy was inconsistently recorded throughout the studies. There is no standard in the duration of iron supplementation, occurring over different periods of pregnancy. 23 26 27 29 30 Three studies did not report the duration of iron supplementation. 24 25 28 Blood collection for hemoglobin and/or ferritin tests was performed at different times, during the first, 24 25 28 30 second, 24 25 27 28 and third trimesters, 13 28 as well as at delivery. 23 It was also performed at first visits, and at 12, 20, 28, and 36 weeks of gestation. 29

The diagnosis of GDM was measured with an oral glucose tolerance test (OGTT) in 3 studies: in 3, the test was done at 24 to 28 weeks, 24 25 26 and in 1 at 28 to 30 and 36 weeks. 13 Furthermore, 3 studies did not present any information on GDM diagnosis 23 28 30 and 2 mentioned that the diagnosis was extracted from patients' medical record. 27 29 The diagnosis of GDM was performed between 24 and 28 weeks of gestation in 3 of the studies; 24 25 26 Rawal et al. 27 reported it at 10 to 14 and 15 to 26 weeks; Chan et al. 13 reported it at 28 to 30 and 36 weeks; and Liu and Pang 23 reported it at delivery. Finally, 3 studies did not report the time of GDM diagnosis. 28 29 30

Among the RCTs, none of them found a significant difference in the incidence of GDM between women in the control and experimental groups ( Chart 3 ). 13 29 30 On the other hand, the non-randomized clinical trial (NRCT) found an association between iron supplementation in pregnant women with normal ferritin levels and the increased risk of GDM. 28

**Chart 3:** Quantitative results from controlled trials

First author (year)	Biomarker of iron		Trimester collected	Outcomes (incidence of GDM)		Statistically significant difference
	Hemoglobin (g/dL)	Ferritin (pmol/L)		Control group	Experimental group	
Asadi (2019) 28	Control group 1: 1 ^st^ 12.24 ± 1.5 2 ^nd^ 11.88 ± 1.4 3 ^rd^ 12.39 ± 2.29 Control group 2: 1 ^st^ 12.54 ± 1.23 2 ^nd^ 12.02 ± 1.06 3 ^rd^ 12.46 ± 1.12 Experimental group: 1 ^st^ 14.75 ± 1.42; *p* = 0.452 2 ^nd^ 12.32 ± 1.27; *p* = 0.387 3 ^rd^ 12.79 ± 1.18; *p* = 0.59	NR	1 ^st^ , 2 ^nd^ , and 3 ^rd^	0 (0%) 0 (0%)	5 (16.7%)	Yes *p* = 0.038
Chan (2009) 13	Control group ( *n* = 531) 11.4 ± 0.04 Experimental group ( *n* = 511) 11.4 ± 0.04 p < 0.001	Control group ( *n* = 490) 49.14 ± 2.17 Experimental group ( *n* = 469) 60.4 ± 2.22 p < 0.001	3 ^rd^	−	OR = 1.04 CI = 0.7–1.53	No
Kinnunen (2016) 29	Control group: 20 weeks 126.4 ± 8.9 28 weeks 123.9 ± 9.5 36 weeks 124.8 ± 9.6 Experimental group: 20 weeks 127.0 ± 8.6; *p* = 0.11 28 weeks 127.0 ± 8.6; *p* < 0.001 36 weeks 131.2 ± 9.3; *p* < 0.001	NR	2 ^nd^ and 3 ^rd^	11.0%	13.0%	No *p* = 0.12
Ouladsahebmadarek(2011) 30	Control group: 12.48 ± 0.91 Experimental group: 13.46 ± 0.75; *p* = 0.03	Control group (μg/dl): 9.26 ± 0.62 Experimental group (μg/dl): 26.91 ± 2.11; *p* = 0.048	At delivery	0.8%	0.5%	No *p* = 0.67

**Abbreviations:** CI, confidence interval; GDM, gestational diabetes mellitus; NR, not related; OR, odds ratio.

Among the cohort studies ( Chart 2 ), two found no significant associations between iron supplement use and risk of GDM. 23 25 Si et al. 24 verified that the iron supplementation in pregnant women with high hemoglobin concentration (Hb > 11) increased the risks for GDM. Among the case-control studies, one used average hemoglobin and ferritin, 26 and another used only ferritin. 27 The average hemoglobin and ferritin levels were higher in women with GDM in both studies ( Chart 4 ).

**Chart 4:** Quantitative results from observational studies

First author, year	Biomarker of iron		Trimester collected	OR crude (95% CI or p-value)	OR adjusted (95% CI)	Control for confounding
	Hemoglobin (g/dL)	Ferritin (pmol/L)				
Özyiğit (2008) 26	Iron group 12.3 ± 1.3 Control group 12.1 ± 1.04	Iron group 16.5 ± 6.09 Control group 12.5 ± 9.7 (ng/mL) ( *p* = 0.018)	1 ^st^	−	−	BMI, age, parity.
Rawal (2016) 27	NR	GDM group 94.5 (67.9–133.5) No GDM group 78.1 (49.7–119.4)	2 ^nd^	3.06 (CI = 1.27–7.34) 15–26 weeks; highest vs. lowest quartiles	**3.95 (CI= 1.38–11.3)** 15–26 weeks; highest vs. lowest quartiles	BMI, parity, education, family history of diabetes.
Liu and Pang (2018) 23	Iron group 12.1 ± 0.06 Control group 11.7 ± 0.08 p <0.01	Iron group 65.8 ± 3.1 Control group 53.7 ± 2.9 p <0.01	At delivery	1.02 ( *p* = 0.96)	−	NR
Zhu (2019) 25	GDM group 1 ^st^ 128.8 ± 9.2 2 ^nd^ 115.4 ± 9.6 No GDM group 1 ^st^ 127.2 ± 9.3 2 ^nd^ 114.6 ± 9.9 (g/L)	NR	1 ^st^ and 2 ^nd^	1 ^st^ = 1.1 (1.04–1.16) 2 ^nd^ = 1.04 (0.99–1.1)	1 ^st^ ( *n* = 113) Crude model = 1.19 (0.94–1.49) Model 1 = 1.12 (0.88–1.43) 2 ^nd^ ( *n* = 63) Crude model = 1.13 (0.85–1.52) Model 1 = 1.06 (0.78–1.44)	Age, prepregnancy BMI, family history of diabetes, parity, smoking, drinking, diastolic blood pressure, systolic blood pressure, gestational week at visit, income, and education.
Si (2020) 24	GDM group 1 ^st^ 12.68 ± 0.91 2 ^nd^ 11.57 ± 0.83 No GDM group 1 ^st^ 12.54 ± 0.88 2 ^nd^ 11.43 ± 0.83	NR	1 ^st^ and 2 ^nd^	−	IS in 1 ^st^ / Hb in 2 ^nd^ trimester Hb <11 ( *n* = 80) 1.32 (0.67–2.51) Hb >11 ( *n* = 217) **1.53 (1.05–2.24)** IS in 2 ^nd^ / Hb in 2 ^nd^ trimester Hb <11 ( *n* = 184) 1.23 (0.68–2.28) Hb >11 ( *n* = 305) **1.92 (1.13–3.35** ) IS in 2 ^nd^ / Hb in 1 ^st^ trimester Hb < 11 ( *n* = 54) 1.04 (0.13–2.21) Hb > 11 ( *n* = 435) **2.15 (1.07–4.34)**	Maternal age, education, pre-pregnancy BMI, parity, weight gain during pregnancy, dietary iron intake, family history of diseases, isolated hypothyroxinemia in 1 ^st^ trimester, participation in physical exercise during pregnancy, gestational week of Hb measurement and OGTT measurement.

**Abbreviations:** BMI, body mass index; CI, confidence interval; IS, iron supplementation; NR, not reported; OGTT, oral glucose tolerance test; OR, odds ratio; RR, relative risk.

**Fig. 2:**
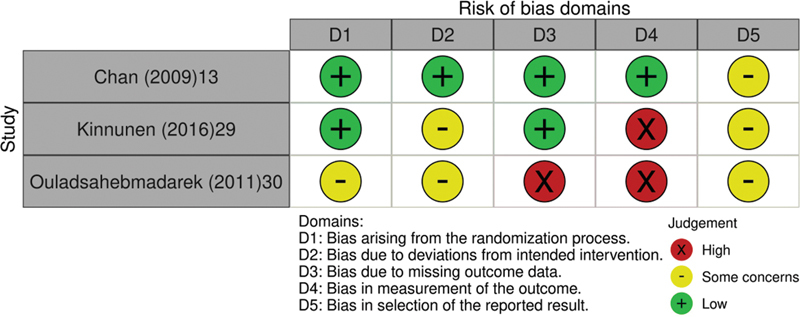
Graphical representation of risk of bias assessment of the Randomized Clinical Trials included in the review ( *n* = 3). Source of bias risk assessment: Cochrane Collaboration tool.

Based on ROB-2, no domain was classified as low risk of bias for all randomized clinical trial evaluated; 2 of the studies presented high risk of bias in relation to measurement of the outcome, 29 30 and 1 study presented high risk of bias due to missing outcome data. 30 ( Fig. 2 ) Regarding the NRCT, the evaluation based on ROBINS-I classified the study as serious risk of bias due confounding and classification of interventions, and low risk of bias in selection of participants into the study and deviations from intended interventions. No information was reported on missing data and measurement of outcomes to allow the evaluation of bias on these domains. 28

**Fig. 3:**
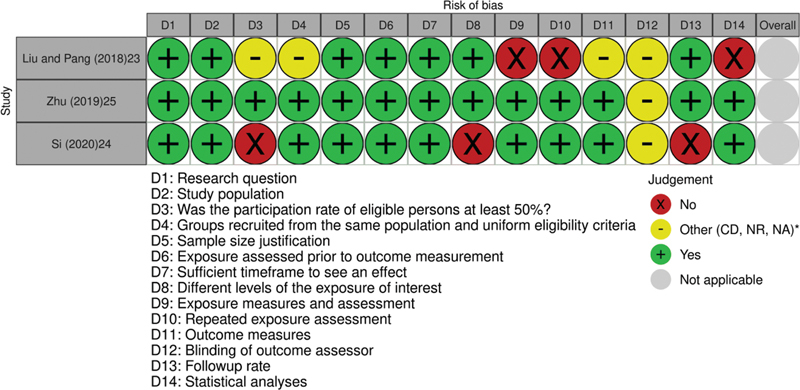
Graphical representation of risk of bias assessment of the Cohort Studies included in the review ( *n* = 3). Source of quality assessment: National Institutes of Health (NIH). *Abbreviations: CD, cannot determine; NR, not reported; NA, not applicable.

**Fig. 4:**
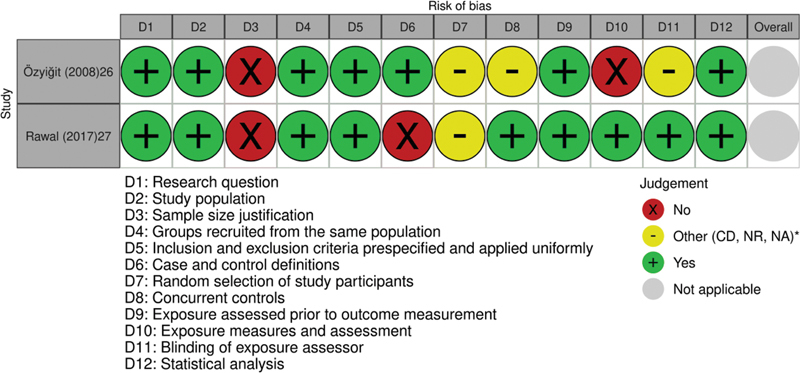
Graphical representation of risk of bias assessment of the Case-Control Studies included in the review ( *n* = 2). Source of quality assessment: National Institutes of Health (NIH). *Abbreviations: CD, cannot determine; NR, not reported; NA, not applicable.

Based on the NIH tool, cohort studies had a low risk of bias for most of the questions, mainly the study of Zhu et al. 25 Liu and Pang 23 did not clearly define their exposure measures, did not realize adjustments considered important in the analyses, such as parity and maternal age, and presented a high risk for evaluation of repeated exposure, that is, were not performed multiple measurements of the hemoglobin and/or ferritin, which would provide greater confidence that the exposure status was correctly classified. 23 Si et al. 24 did not provide information on loss of follow-up after the baseline and, presented high risk in different levels of the exposure of interest and the participation rate at least 50% of eligible persons either ( Fig. 3 ). In the case-control studies, Özyiğit et al. 26 selected the study sample from exposure to the use of iron, and Rawal et al. 27 did not have clearly defined and differentiated cases of controls; furthermore, both studies did not report a sample size justification. 26 27 ( Fig. 4 )

## Discussion

A systematic review with a robust search strategy was conducted to evaluate the association between iron supplementation, hemoglobin levels and/or serum ferritin, and the risk of developing GDM. There were few studies of which only three were RCTs with exposure to the use of iron supplementation and information on iron biomarkers (ferritin and/or hemoglobin).

The association investigated in this review has biological plausibility. Iron has a high oxidation capacity, and its free form can catalyze the formation of free radicals, which may lead to cellular damage, also known as oxidative stress. It is known that pregnancy by itself is a condition that favors the occurrence of oxidative stress because the placenta is rich in mitochondria, as well as for the fact that the transition metals, especially iron, are particularly abundant in the placenta, which would already boost the production of free radicals. 11 Thus, one of the suggested mechanisms involves the formation of the hydroxyl radical, which can damage β-cell membranes, affecting the synthesis and secretion of insulin by the pancreas. 11

Another proposed mechanism would be more peripheral. The deposition of iron in the muscle would damage the muscle tissue, which would consequently lead to a decrease in glucose uptake. Additionally, insulin would be responsible for stimulating iron uptake by the cell, which could lead to an even greater accumulation of cellular iron, forming vicious cycle that could induce insulin resistance and diabetes mellitus. 31 32

Most studies included in our review did not find a positive association between iron supplementation during pregnancy and GDM. We did find a positive association between hemoglobin or ferritin levels and increased risk of developing GDM, based on cohort and case-control studies; however, the same association was not found in RCTs. The positive association found in the case-control studies 26 27 may reflect the condition in which the increased ferritin in GDM was, as it is an acute phase reactant and the increased levels seen in women with GDM were a result of the inflammation associated with the disease. 13

Among the reasons for the differences found in the types of studies, the methodological aspects stand out; RCT is a type of study very similar to prospective cohort studies, with the difference being that its design makes it possible to remove several biases, such as confounding and selection bias, since the treatment and control groups are allocated using random techniques and the characteristics are similarly distributed in both groups. However, the RCTs included in this review presented methodological problems that may have compromised their ability to assess the causality between iron supplementation, elevated hemoglobin and ferritin, and the development of GDM. Among the problems found, stands out the bias in the measure of GDM. This flaw was detected in nonrandomized studies too. Some studies did not report what was considered for diagnosis, 23 28 30 and among those that did, some used the values of the OGTT test performed between the 24 ^th^ and 28 ^th^ gestational week for the diagnosis of GDM, 24 25 26 others assessed OGTT at different gestational weeks, 13 27 or considered other criteria, such as investigating GDM only in pregnant women with risk factors. 29 These inconsistencies regarding the type of method and screening period for the diagnosis of GDM may cause misclassification bias.

In the NRCT, there was not enough information regarding missing data and measurement of outcomes to classify as serious or critical risk of bias. The measure of the diagnostic of GDM and the initial sample as well as the missing data were not related. 28

Regarding the case-control studies, the selection bias was the most important one, mainly regarding the representativeness. Information about sample size justification 26 27 and a specific description of case and control groups was not provided. 27 Furthermore, one of the included studies selected the cases and controls regarding the exposure. 26 Among cohort studies, although Si et al. 24 showed a positive association between iron supplementation and GDM in women with Hb > 11, some methodological flaws cause concerns regarding the rate of eligible persons and the follow-up baseline being less than 20%.

Other limitations related to the revised data and the review process must be considered. Regarding the reviewed studies, we identified that there was no standard for the hemoglobin and/or ferritin collection in the trimester of pregnancy, which did not allow us to evaluate the dose–response effect of these parameters on the use of iron supplementation. The time of use, period of onset, and dosage of iron supplement were also different among the studies evaluated. Despite this review only including studies that assess ferritin and/or hemoglobin, two studies reported information on dietary iron intake. 13 24 There is a demand for studies that assess multiple factors that could influence iron status in pregnant women. Distinguishing the origin of iron, whether diet or supplementation, is a limiting factor. Thus, studies including the use of other parameters to assess the iron status and evaluated stores, supplementation, and dietary iron intake, as well as its role in the development of GDM are required.

Although we performed a comprehensive review, including various databases, gray literature, and with no language restriction, a possible publication bias cannot be ruled out. On the other hand, this review, unlike other existing ones, 33 34 used as in inclusion criterion the presence of at least one hematological parameter of iron (hemoglobin and/or ferritin), commonly required during antenatal care and, as main exposure, the use of iron through supplements. Additionally, this is the only review on the topic that has included observational and interventional studies.

## Conclusion

Based on the studies included in this review and the critical methodological problems, the association between iron supplementation and GDM remains undetermined. Therefore, this review highlights the necessity for more studies evaluating the relationship between iron supplementation and GDM.
